# The sero‐epidemiology of *Coxiella burnetii* (Q fever) across livestock species and herding contexts in Laikipia County, Kenya

**DOI:** 10.1111/zph.12567

**Published:** 2019-02-20

**Authors:** Peter S. Larson, Leon Espira, Cole Grabow, Christine A. Wang, Dishon Muloi, A. Springer Browne, Sharon L. Deem, Eric M. Fèvre, Johannes Foufopoulos, Rebecca Hardin, Joseph N. S. Eisenberg

**Affiliations:** ^1^ School for Environment and Sustainability University of Michigan Ann Arbor Michigan; ^2^ Department of Epidemiology, School of Public Health University of Michigan Ann Arbor Michigan; ^3^ Department of Population Health and Pathobiology, College of Veterinary Medicine North Carolina State University Raleigh North Carolina; ^4^ Centre for Immunity, Infection and Evolution University of Edinburgh Edinburgh UK; ^5^ International Livestock Research Institute Nairobi Kenya; ^6^ Usher Institute of Population Health Sciences and Informatics University of Edinburgh Edinburgh UK; ^7^ Saint Louis Zoo Institute for Conservation Medicine Saint Louis Missouri; ^8^ Institute of Infection and Global Health University of Liverpool Liverpool UK

**Keywords:** Kenya, land management, livestock economy, Q fever, seroepidemiology, zoonosis

## Abstract

*Coxiella burnetii*, the causative agent of Query fever (Q fever), is among the most highly infectious zoonotic pathogens transmitted among livestock, with chronic effects challenging to veterinary and medical detection and care systems. Transmission among domestic livestock species can vary regionally due to herd management practices that determine which livestock species are raised, whether or not livestock are in contact with wildlife, and the susceptibility of these livestock to infection. To explore how different livestock management practices are associated with the risk of infection in multispecies environments, we carried out a comparative study of three types of herd management systems in the central Kenyan county of Laikipia: agro‐commercial, mixed conservancy/commercial, and smallholder ranches. We tested *C. burnetii* antibody seroprevalence in four common livestock species. Across all management types, the highest seroprevalence was in camels (20%), followed by goats (18%), sheep (13%), and cattle (6%). We observed a lower odds of testing seropositive for young compared to adult animals (adjusted OR = 0.44 [95% CI 0.24, 0.76]), and for males compared to females (adjusted OR = 0.52 [95% CI 0.33, 0.80]). Animals from mixed conservancy/commercial and smallholder operations had a higher odds of testing seropositive compared to animals from agro‐commercial ranches (adjusted OR = 5.17 [95% CI 2.71, 10.44] and adjusted OR = 2.21 [95% CI 1.17, 4.43] respectively). These data suggest that herd management practices might affect the transmission dynamics of *C. burnetii*in arid African ecosystems like those seen in Kenya where several transmission modes are possible, risk of drought has promoted new livestock species such as camels, and multiple wildlife species may co‐occur with livestock on the landscape. Further longitudinal studies are needed to disentangle the mechanisms underlying these patterns, and further explore transmission patterns between wildlife, domestic animal, and human populations.

**Impacts**

- Camels, whose presence as livestock increased relatively recently (early 1990s), were more likely to test seropositive for *Coxiella burnetii* than the more established cattle populations.
- Animals from sites of mixed wildlife and livestock, and as well as smallholder operations, had higher odds of testing seropositive for *C. burnetii* than animals from agro‐commercial operations (independent of sex and age distributions).
- Herd management practices have a significant impact on the transmission dynamics of *C. burnetii*, especially when several transmission modes are possible, a new species is introduced into the region, and livestock co‐occur with wildlife.

## 1 INTRODUCTION

*Coxiella burnetii*, the causative agent for Query fever (Q fever), is a zoonotic pathogen transmitted among humans and livestock. In recent years, there has been an increased awareness of Q fever as an economically important pathogen due to a rise in the frequency of reported outbreaks and the economic impact of Q fever has on commercial livestock operations in the form of lost animal reproductive productivity and herd death (Enserink, 2010; Ziva et al., 2010). Important epidemiological factors that aid in the transmission of *C. burnetii* and make control efforts difficult include its: (a) high transmissibility, (b) ability to infect and transmit among multiple mammalian host taxa along multiple pathways and (c) persistence over long periods of time in the environment (Gilbert et al., 2013; Maurin & Raoult, 1999; Raoult, Marrie, & Mege, 2005). Although *C. burnetii* can spread vertically to offspring, horizontally through contact with bodily fluids, or by arthropod vectors (Maurin & Raoult, 1999) the primary mode of infection is thought to be through inhalation of aerosolized bacteria (Raoult et al., 2005) shed into the environment along with birth products, urine, faeces and other fluids. Once in the environment, the bacteria can persist for weeks, months or even longer (Gilbert et al., 2013; Maurin & Raoult, 1999). Due to the multihost potential of the pathogen, control may be difficult in areas where livestock and wildlife interact.

In addition to biological transmission factors, herd management practices also play a role in the epidemiology of *C. burnetii*. For example, in East Africa, the livelihoods of pastoralists have historically depended on being able to range over wide areas with livestock in resource‐limited landscapes (Catley, Lind, & Scoones, 2013). The ability to range long distances helped ensure adequate grazing opportunities for livestock, and possibly allowed pastoralists to minimize contact with infected herds. Following Kenya's independence in 1963, livestock and herd management systems changed. Many pastoralists were forced to move towards pseudo‐sedentary herding practices or adopt strategies that mix agriculture and smallholder livestock herding. Modern commercial ranching operations with herd management practices based on managing cattle by limiting and rotating grazing areas, producing feed on site, and the use of targeted strategies such as the regular administration of medications and culling to insure herd health and to maximize productivity have become more common. Many of these commercial ranches, however, maintain some traditional pastoral practices and often commercial ranches and modern day pastoral communities coexist alongside one another. This has led to a mosaic of land and livestock management practices each with their own potentially distinct risk of disease transmission.

The central Kenyan county of Laikipia is an example of an area with such a mosaic, making it amenable to the design of field experiments for improved understanding of this complexity. Laikipia is a major economic pillar of Kenyan's large livestock production industry (Food & Agriculture Organization of the United Nations, 2017). Colonial era ranches established by expatriates and Kenyans of European descent now sit abreast with independence era “group ranches” accorded around 1960 to local communities. African Kenyan‐owned ranches purchased since independence are also now common. More recently, new industries have arisen such as tourism and research that complement the still dominant and profitable ranching operations that produce meat and dairy. Laikipia's livestock trade includes traditional species such as goats, sheep, cattle; but increasingly also camels, which have been imported in response to changing rainfall patterns (Kagunyu & Wanjohi, 2014).

The diverse ecological profile of Laikipia includes a wide variety of wild ungulates such as zebras or antelopes that share grazing lands with domesticated livestock, potentially increasing or limiting opportunities for transmission of pathogens from wildlife to livestock (Odadi, Jain, Wieren, Prins, & Rubenstein, 2011). Sustained inter‐wildlife transmission and the pathogen's ability to persist for prolonged periods in the environment make controlling Q fever difficult for livestock keepers. Here we aim to further the understanding of the seroepidemiology of *C. burnetii* across multiple livestock species in an ecologically diverse area of Kenya with a multitude of livestock and herd management systems.

## 2 MATERIALS AND METHODS

### 2.1 Study site

Laikipia County is located ~250 km north of Nairobi on the Laikipia plateau. The Laikipia plateau ranges in elevation from 1,700 to 2,550 m, bordered by Mount Kenya to the southeast and the Great Rift Valley to the west. Mean temperatures range from 22 to 26°C depending on season and elevation (District of Laikipia, 2007). Though the landscape in Laikipia varies in terms of vegetative cover and availability of water, it is an arid region with two short rainy seasons, but like all of East Africa, has experienced inconsistent rains and periods of extreme drought in recent years.

### 2.2 Herd management in Laikipia and sampling areas

Prior to beginning data collection, we conducted interviews with property and livestock owners, animal handlers and staff. From these exploratory interviews, we opted to divide livestock management in Laikipia into three rough categories of land and animal management: (a) agro‐commercial ranches, (b) mixed wildlife conservancies and livestock operations and (c) smallholder animal herding. We noted differences for each in domesticated herd species composition, chemical and veterinary medicine use patterns, and exclusion of livestock from wildlife through practices like fencing. Agro‐commercial ranches produce their own feed and actively manage grazing behaviours and animal health to maximize commercial productivity in what are often cattle dominated or even single species herds with high rates of chemical dipping to minimize infestations by ectoparasites such as ticks. Mixed wildlife conservancies and livestock operations allow or encourage the presence of wildlife on their lands, but also set aside spaces for commercial livestock, often in species‐exclusive herds, and with moderate access to chemical and veterinary products. Smallholder animal operations have smaller, herds that range in areas that allow herders to travel to and from their homes in a single day. These households often focus on small ungulates but sometimes include mixed herds of cattle, sheep and goats. While they show relatively high understanding of chemical and veterinary products in relation to pathogens they have limited capital available to invest in pharmaceuticals to prevent or treat disease outbreaks (Browne et al., 2017). Thus, the three categories are general proxies for a variety of approaches to animal management and the context within which animals are raised.

**Figure 1:**
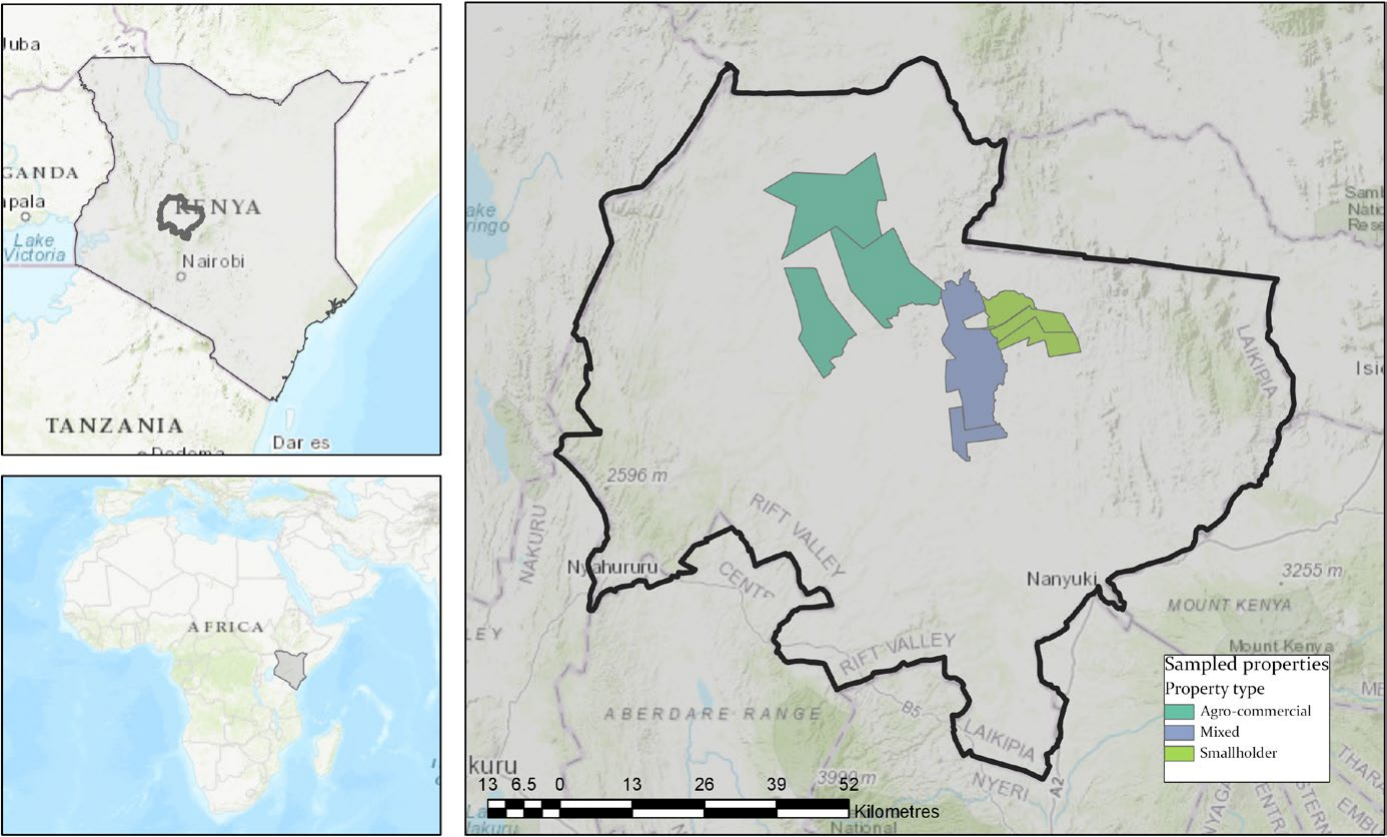
Map of the sampled study sites and their classification within Laikipia County (outlined in black), Kenya. The green polygons represent agro‐commercial ranching operations; the blue represent wildlife and livestock operations; and the olive represent small holder animal herding areas [Colour figure can be viewed at wileyonlinelibrary.com]

We examined the impact of these three different livestock management types, agro‐commercial, mixed wildlife and livestock, and smallholder herding, on *C. burnetii* antibody seroprevalence in cattle, camels, goats and sheep using serum samples collected from nine sites in Laikipia County, Kenya. We considered four sites as agro‐commercial ranches, three as mixed wildlife conservancies and livestock operations and two as smallholder livestock herds (Figure 1). This extends our previous work on camels in this region (Browne et al., 2017; DePuy et al., 2014) by examining *C. burnetii* seropositivity across multiple livestock species and management types.

### 2.3 Sero‐sampling of livestock and interviews

Research activities occurred in cooperation with local partners from June to August 2013. We conducted stratified random sampling aiming for at least 15% of all animals of each species from each livestock herd (for herds > 20 animals). For small herds (<20 animals), every animal was considered for inclusion. Attempts were made to evenly represent each age group and to represent the sex distribution of each herd; however, adult male cattle and camels were under‐sampled due to safety concerns. Animals were assigned one of three age categories, young (≤6 months), juvenile (6 months–2 years) and adult (>2 years), based on visual assessment and information obtained from livestock managers or lead herders. With the cooperation of ranch staff, the team, which included licensed Kenyan veterinarians, obtained 4–8 ml of blood from each animal using an 18‐gauge needle with a 10 ml syringe. Blood was then placed into an 8.5 ml serum separator tube. Samples were stored with ice packs for transport between the sampling sites and the Mpala Research Centre. Within 6 hr of collection, samples were centrifuged for 10 min and aliquots of 1–3 ml serum per sample were placed into cryotubes and stored in a −20°C freezer for transport to the International Livestock Research Institute (ILRI) in Nairobi. All sera were analysed for *C. burnetii* IgG antibodies using the CHECKIT Q Fever Antibody ELISA Test Kit (IDEXX Europe B.V, The Netherlands) as per the manufacturer's instructions. ELISA assays were carried out at ILRI and the Nagasaki University Institute for Tropical Medicine‐Kenya Medical Research Institute's P3 laboratory in Nairobi, Kenya.

### 2.4 Outcome measure, exposure measure and covariates

Our outcome variable is seroprevalence, the proportion of animals that tested positive for IgG antibodies to *C. burnetii*. Seroprevalence is a measure of past infection. Our main exposure variable is the livestock management type at a particular property; agro‐commercial, mixed wildlife conservancies and livestock operations, and smallholder herding. Other covariates in our analysis include animal age and sex, both of which have been reported as risk factors for *C. burnetii* seroprevalence in Laikipia and in other parts of Africa (Browne et al., 2017; Mazeri et al., 2013; Scolamacchia et al., 2010).

### 2.5 Data management/analysis

We first assigned each animal a unique identifier based on the herd identifier, the species of the animal and the order of sampling. We noted identifiers both in a paper logbook and on the tubes used for sero‐sampling. We recorded site name, time and GPS location in the logbook along with sex, age, body condition and relevant notes. We assessed the tick burden of all animals by examining the groin, axilla, perineum and ears and assigned each animals to either no ticks, 0–100 ticks, or 100+ ticks (Browne et al., 2017). A veterinarian assessed females for pregnancy status. All data were entered into a digital database daily. Descriptive statistics were produced for herd demographics, seroprevalence and health information. We used bivariate logistic regression to test associations of *C. burnetii* seropositivity with relevant predictors (species, sex, age, pregnancy status, herd management type and tick load). We produced a multivariate model of *C. burnetii* seropositivity from the available data and a backward selection procedure based on Akaike's Information Criterion (AIC). All analyses were carried out using r version 3.4.3 (R Core Team, 2018).

### 2.6 Ethical approval

Ethical approval for sample collection was granted by the Kenyan National Council of Science and Technology (NCST; permit number NCST/RRI/12/1/BS011/064), the University of Michigan University Commission on the Use and Care of Animals (UCUCA), and the University of Michigan Institutional Review Board (IRB). We obtained informed consent from livestock owners and property managers.

## 3 RESULTS

### 3.1 Demographic information/data

We tested 849 animals (157 cattle, 312 camels, 280 goats and 100 sheep) for the presence of antibodies to *C. burnetii* (Table 1). Camels were the most likely to be seropositive (19.9% [95% CI 15.4%, 24.3%]) for *C. burnetii*, followed by goats (18.2% [95% CI 13.7%, 22.7%]) and sheep (13.0% [95% CI 6.4%, 19.6%]). Cattle were the least likely to be seropositive (5.7% [95% CI 2.1%, 9.4%]). Most sampled animals from each species were female (63.9%). Overall, the majority of sampled animals were adults (56.5%), with some species‐specific variation. We assessed pregnancy status only in goats and sheep, with more than half of the females in each species assessed as being pregnant (58.5% and 58.2%, respectively). A little over half of the sampled animals were from smallholder operations (54.9%), reflecting that most of the goats (87.9%) and sheep (70.1%) that made up almost half the sampled livestock (46.3%) were kept in smallholder operations.

**Table 1:** Demographics of the aggregated ranching operations (categorized into three groups). A total of 990 animals were sampled across the three herd management types and 849 animals had their *Coxiella burnetii* sero‐status determined. Sex and age were determined for 986 animals, while tick scores were available for 661 animals

Variable	[ALL] *N* = 849	Bovine *N* = 157	Camel *N* = 312	Goat *N* = 280	Sheep *N* = 100	*p*‐Value
*C. burnetii* sero‐status						
Negative	714 (84.1%)	148 (94.3%)	250 (80.1%)	229 (81.8%)	87 (87.0%)	0.001
Positive	135 (15.9%)	9 (5.73%)	62 (19.9%)	51 (18.2%)	13 (13.0%)	
Sex						
Female	630 (63.9%)	126 (65.3%)	218 (65.1%)	207 (64.5%)	79 (57.7%)	0.437
Male	356 (36.1%)	67 (34.7%)	117 (34.9%)	114 (35.5%)	58 (42.3%)	
Age category						
Adult (>2 years)	557 (56.5%)	80 (41.5%)	198 (58.9%)	181 (56.6%)	98 (71.5%)	<0.001
Juvenile (6 months–2 years)	220 (22.3%)	46 (23.8%)	81 (24.1%)	74 (23.1%)	19 (13.9%)	
Young (<6 months)	209 (21.2%)	67 (34.7%)	57 (17.0%)	65 (20.3%)	20 (14.6%)	
Pregnancy status						
N	167 (58.4%)	NA	NA	121 (58.5%)	46 (58.2%)	1.000
Y	119 (41.6%)	NA	NA	86 (41.5%)	33 (41.8%)	
Property type						
Agro‐Commercial	167 (16.9%)	23 (11.7%)	114 (33.9%)	22 (6.85%)	8 (5.84%)	<0.001
Mixed	279 (28.2%)	107 (54.6%)	122 (36.3%)	17 (5.30%)	33 (24.1%)	
Smallholder	544 (54.9%)	66 (33.7%)	100 (29.8%)	282 (87.9%)	96 (70.1%)	
Tick score						
0	440 (66.6%)	109 (97.3%)	147 (43.9%)	124 (86.1%)	60 (85.7%)	<0.001
1	200 (30.3%)	3 (2.68%)	167 (49.9%)	20 (13.9%)	10 (14.3%)	
2	21 (3.18%)	0 (0.00%)	21 (6.27%)	0 (0.00%)	0 (0.00%)	

### 3.2 Bivariate analysis

Bivariate analyses were conducted to evaluate whether seroprevalence differed by species, age, sex, property management type and tick burden (Table 2). The odds of seropositivity for camels were more than four times higher (OR = 4.01 [95% CI 2.03, 8.92]) than for cattle. Similarly, the odds of testing seropositive were nearly four times higher for goats compared to cattle (OR = 3.60 [95% CI 1.80, 8.09]) and similarly higher for sheep compared to cattle (OR = 2.44 [95% CI 1.00, 6.20]). For all species, males were less likely to be seropositive than females (OR = 0.53 [95% CI 0.35, 0.80]). Juvenile and young animals had significantly decreased odds of testing seropositive when compared with adult animals (OR = 0.48 [95% CI 0.29, 0.77] and OR = 0.38 [95% CI 0.21, 0.64], respectively).

**Table 2:** Bivariate analysis using seropositivity among surveyed animals as the outcome variable. Statistically significant results are bolded

Variable	Negative *N* = 715	Positive *N* = 135	Odds Ratio
Species			
Bovine	148 (20.7%)	9 (6.67%)	Ref.
Camel	250 (35.0%)	62 (45.9%)	**4.01 [2.03;8.92]**
Goat	229 (32.1%)	51 (37.8%)	**3.60 [1.80;8.09]**
Sheep	87 (12.2%)	13 (9.63%)	**2.44 [1.00;6.20]**
Sex			
Female	429 (60.3%)	100 (74.1%)	Ref.
Male	283 (39.7%)	35 (25.9%)	**0.53 [0.35;0.80**]
Age category			
Adult (>2 years)	360 (50.5%)	95 (70.4%)	Ref.
Juvenile (6 months–2 years)	182 (25.5%)	23 (17.0%)	**0.48 [0.29;0.77]**
Young (<6 months)	171 (24.0%)	17 (12.6%)	**0.38 [0.21;0.64]**
Property type			
Agro‐Commercial	142 (19.9%)	14 (10.4%)	Ref.
Mixed	160 (22.4%)	48 (35.6%)	**3.01 [1.63;5.91]**
Smallholder	413 (57.8%)	73 (54.1%)	**1.78 [1.00;3.38]**
Tick score			
0	336 (68.4%)	62 (58.5%)	Ref.
1	141 (28.7%)	38 (35.8%)	1.46 [0.93;2.28]
2	14 (2.85%)	6 (5.66%)	2.35 [0.79;6.17]
Pregnant			
N	111 (61.3%)	29 (58.0%)	Ref.
Y	70 (38.7%)	21 (42.0%)	1.15 [0.60;2.17]

Property/management type was associated with the odds of *C. burnetii*seropositivity. Compared with agro‐commercial operations, animals owned by ranching operations that included and encouraged the presence of large wildlife species had three times higher odds of seropositivity (OR = 3.01 [95% CI 1.63, 5.91]). Animals in smallholder ranching areas had almost two times higher odds of being seropositive compared to agro‐commercial operations (OR = 1.78 [95% CI 1.00, 3.38]). Higher tick loads resulted in elevated point estimates for the odds of being seropositive for *C. burnetii*, but this relationship was non‐significant. Pregnancy was also not associated with seropositivity.

### 3.3 Multivariate analysis

We tested for within‐herd correlation and concluded that a mixed model including a random effect for herd was not required. We excluded tick score from the model given the large number of missing observations and pregnancy status since it was not significant in the bivariate analysis. We also tested model performance against other possible models from subsets of the variables included and with interaction terms (e.g., species, age). Our final model, which represented the best possible model from the variables at hand, included animal species, sex, age and site type.

Even in the presence of confounders, camels and goats were found to have more than five times higher odds of being seropositive for *C. burnetii* compared with cattle (adjusted OR = 5.26 [95% CI 2.56, 12.01] and adjusted OR = 5.54 [95% CI = 2.49, 13.55], respectively; Table 3). Sheep were less likely to test positive compared to cattle than either camels or goats compared to cattle (adjusted OR = 3.38 [95% CI 1.28, 9.29]). As in the bivariate analysis, males were less likely to be seropositive for *C. burnetii* than females (adjusted OR = 0.52, [95% CI 0.33, 0.80]) as were juvenile and young animals, who had decreased odds of being seropositive (adjusted OR = 0.53 [95% CI 0.31, 0.87] and adjusted OR = 0.44 [95% CI 0.24, 0.76], respectively). Even when accounting for confounders of species, age and sex, animals from sites of mixed wildlife and livestock operations were more than five times more likely to be seropositive for *C. burnetii* than animals in agro‐commercial contexts (Adjusted OR = 5.17 [95% CI 2.71, 10.44]). Further, the odds of seropositivity for animals in smallholder contexts were twice as high as for animals in agro‐commercial contexts (Adjusted OR = 2.21 [95% CI 1.17, 4.43]).

**Table 3:** Multiple logistic regression analysis

Variable	Odds ratio (95% CI)
(Intercept)	0.03 (0.01, 0.07)
Bovine (ref.)	
Camel	5.26 (2.56, 12.01)
Goat	5.54 (2.49, 13.55)
Sheep	3.38 (1.28, 9.29)
Sex	
Female (ref.)	
Male	0.52 (0.33, 0.8)
Age category	
Adult (>2 years) (ref.)	
Juvenile (6 months–2 years)	0.53 (0.31, 0.87)
Young (<6 months)	0.44 (0.24, 0.76)
Property type	
Agro‐commercial (ref.)	
Mixed	5.17 (2.71, 10.44)
Smallholder	2.21 (1.17, 4.43)

## 4 DISCUSSION

*Coxiella burnetii* seroprevalence varied by animal species in Laikipia, Kenya, consistent with our prior publication (Depuy et al., 2014). The low seroprevalence among cattle was consistent with our study conducted in Laikipia (*C. burnetii* seroprevalence = 4%; DePuy et al., 2014), as well as with a study in the Sahelian region of Chad that sampled cattle from the herds of nomadic Fulani and Arab cattle herders (*C. burnetii* seroprevalence 4%; Schelling et al., 2003). Other studies in sub‐Saharan Africa, however, have reported higher seroprevalence among cattle. For example, in West Africa looking at Fulani cattle herds (Adesiyun, Jagun, Kwaga, & Tekdek, 1985; Adesiyun, Jagun, & Tekdek, 1984), in the Adamawa region of Cameroon (Scolamacchia et al., 2010), in southeastern Ethiopia (Gumi et al., 2013), and in the Central African Republic (Nakouné et al., 2004) reported 32%–55%, 31%, 32% and 14% *C. burnetii* seroprevalence values, respectively. Within Kenya, two studies of cattle reported *C. burnetii* seroprevalence values of 28% and 11% (Knobel et al., 2013; Wardrop et al., 2016), but both of these studies were done in western Kenya in an area ecologically and economically very different from Laikipia.

Our seroprevalence data for goats and sheep were consistent with previously reported results (Hussien et al., 2012; Nahed Ghoneim, 2012; Schelling et al., 2003), though less than a study in Ethiopia, where antibody seroprevalence was found to be as high as 54% (Gumi et al., 2013). For camels, we had previously reported a seroprevalence of 34.7% (95% CI 23.7, 45.7) in the same area (DePuy et al., 2014). Other studies have shown even higher levels of seroprevalence in camels, including the Sahel region (80% seroprevalence; Schelling et al., 2003) and southeast Ethiopia (up to 90% seroprevalence; Gumi et al., 2013).

The heterogeneity of *C. burnetii* seroprevalence among livestock species could indicate that the transmission dynamics of *C. burnetii* are the result of species‐specific physiologic or environmental factors. We found higher seropositive proportions in camels, which could either reflect the long lifespans of camels compared with ruminants or a greater susceptibility to infection. Alternatively, the higher seroprevalence may be due to environmental factors such as high tick loads or greater densities. Goats were more likely than sheep to be seropositive, despite similar lifespans and moving in mixed herds. This could indicate that sheep are genetically less prone to become infected than goats or could represent differences in mobility, foraging or grooming behaviour. It could also be the case that the impact of *C. burnetii*on reproductive outcomes differs between sheep and goats, allowing more resistant animals to survive. Cattle were the least likely to have been infected with *C. burnetii* in the past, perhaps because of high levels of veterinary care. More work comparing *C. burnetii*infection across species should be done to explain this result.

The socioecological context within which livestock are raised likely impact *C. burnetii*transmission dynamics within and between livestock herds. One review highlighted the importance of understanding exposure risk factors both among livestock populations in the context of African livestock management systems (Vanderburg et al., 2014). In livestock, *C. burnetii* antibody seroprevalence has been associated with age, sex composition, species and proximity to wildlife (Mazeri et al., 2013; Scolamacchia et al., 2010).

A number of factors including differing approaches to animal health may explain the differences in risk for seropositivity between the three types of management systems. Commercial ranches use proactive strategies to maintain herd health. This includes regular application of acaricides to reduce tick burden, the administration of deworming medication and antibiotics and the identification and culling of animals that appear to be sick, all of which may have contributed to a low risk for seropositivity compared with the other types of properties. On the converse, smallholder herders were the most likely to be able to report the health histories of individual animals when requested indicating an awareness of health problems but were the least likely to cull animals when problems arose, fearing the loss of precious financial assets. This might also explain an increased risk for seropositivity compared with commercial contexts. More work should seek to disentangle the specific factors that increase or decrease risk across these three types of animal herding contexts. The small number of properties and herds for this study made a detailed analysis of specific factors difficult.

The level and nature of interaction between livestock and wildlife also vary by management type, which might also affect risks for seropositivity. Agro‐commercial ranches attempt to minimize contact between livestock and wildlife, some actively excluding wildlife through fencing. Conservancies, being areas where wildlife can roam free, offer many opportunities for wildlife of all types to interact with livestock at watering or grazing areas. Smallholder herders have little control over wildlife and livestock interaction outside of that which might occur through reduced levels of vegetation and/or watering spaces, or relatively understudied local hunting practices that likely shape wildlife abundance. We found that animals in areas of mixed wildlife and livestock herding and in smallholder operations were the most likely to have been infected in the past. Contact with wildlife has been shown to be a determinant of similar infections in livestock in other contexts (Dahl & Hjort, 1976). Although our data do not contradict these observations, it is difficult to determine whether contact with wildlife is a contributing factor, since multiple aspects of the epidemiological environment beyond presence of wildlife, change with management strategy. Future research should assess prevalence of Q fever in wildlife species in diverse, wildlife‐rich contexts such as Laikipia.

Environmental factors could influence pathogen transmission. Large commercial livestock operations were situated in areas with higher vegetative cover, higher precipitation, and were proximal to year‐round rivers and watering spaces. Properties with mixed wildlife and livestock activities have moderate access to watering spaces and are located in flat areas suitable to grazing by large animals such as elephants (*Loxodonta africana*) and cape buffalo (*Syncerus caffer*). Smallholder ranchers were often relegated to arid, resource‐poor areas unsuitable for either large‐scale ranching or wildlife conservation. While our limited number of sites prevented a rigorous analysis of the relationship between environmental factors and *C. burnetii* transmission, based on prior studies we speculate that environmental factors are important in determining pathogen persistence and in creating opportunities for transmission.

Studies of cattle have found that proximity to water is a risk for *C. burnetii* infection and that seroprevalence in cattle correlates with precipitation levels (Czaplicki et al., 2012); (Ellen et al., 2009; Wardrop et al., 2016). West African sites reported higher antibody seroprevalence values than drier Sahelian regions (Adesiyun et al., 1985, 1984; Schelling et al., 2003), while wetter western Kenyan sites reported higher antibody seroprevalence values than Laikipia (Knobel et al., 2013; Wardrop et al., 2016). However, under some circumstances dry conditions can also promote infection, as aridity is also associated with aerosolization, which has been found to increase the risk of *C. burnetii*infection in humans and animals (van der Hoek, Hunink, Vellema, & Droogers, 2011; Nogareda et al., 2012; Prabhu et al., 2011; Tissot‐Dupont, Amadei, Nezri, & Raoult, 2004).

Studies have shown that tick exposure might also be a factor in transmission of *C. burnetii*, based on findings that tick infestation is a risk factor for seropositivity (Cantas, Muwonge, Sareyyupoglu, Yardimci, & Skjerve, 2011; van Engelen et al., 2014; Psaroulaki et al., 2006). However, others have suggested that most tick species have low vector capacity to transmit *C. burnetii* and therefore are likely a secondary transmission driver compared to airborne transmission. On the other hand, ticks may play an important role in cross‐species transmission, which can expose *C. burnetii* to different selection pressures that might promote a diversity of virulence and resistance(Duron, Sidi‐Boumedine, Rousset, Moutailler, & Jourdain, 2015). Further work to understand the role of ticks in the transmission of *C. burnetii*between wildlife and livestock and the possibility of other domestic species such as dogs acting as reservoirs of *C. burnetii*is crucial especially in areas where wildlife and livestock co‐occur. Regardless, any analyses of environmental determinants of *C. burnetii* infection in livestock should examine how factors such as the presence of mammalian fauna and livestock management practices intended to prevent or control pathogen infestation may exacerbate or mitigate infection in livestock.

In addition to the economic impact of Q fever in livestock, livestock health directly affects the health of their owners and chronic exposure to zoonotic pathogens serves as a serious health concern for these populations, as well as for those who consume milk or other unpasteurized food products from them (Nyariki, Mwang'ombe, & Thompson, 2009). Understanding human–animal interactions is crucial to both livestock and human disease risk characterization. Human–animal studies need to inform an economic analysis of *C. burnetii* infection on people who are dependent on livestock for their livelihoods and economic growth. Our work here provides information regarding baseline exposure for a human risk analysis. Longitudinal serological studies in both humans and animals can provide a wealth of new information helpful in assessing impacts of Q fever on human health and welfare in places like Kenya.

Kenya, like many lower and middle‐income countries in sub‐Saharan Africa, is undergoing economic changes that have important impacts on human and animal health. The continent‐wide transition from purely nomadic management styles, over semi‐pastoralism to commercial residential ranching has critical implications for the risk of zoonotic transmission within and among different livestock species and to humans. As a result, a more refined understanding of the effects of land management and animal husbandry practices on pathogen transmission within herds will help us identify mechanisms of transmission to inform improved and locally tailored control strategies for infectious disease.

## CONFLICT OF INTEREST

The authors claim no conflicts of interest or competing interests.
